# Development of a Pulsed Xenon Ultraviolet Disinfection Device for Real-Time Air Disinfection in Ambulances

**DOI:** 10.1155/2020/6053065

**Published:** 2020-02-24

**Authors:** Li Song, Wei Li, Jian'an He, Lang Li, Tao Li, Dayong Gu, Huanwen Tang

**Affiliations:** ^1^Department of Environmental and Occupational Health, Dongguan Key Laboratory of Environmental Medicine, School of Public Health, Guangdong Medical University, Dongguan 523808, China; ^2^Central Laboratory of Health Quarantine, Shenzhen International Travel Health Care Center, Shenzhen Customs District, Shenzhen 518033, China; ^3^Shenzhen Pingle Orthopedic Hospital, Shenzhen 518010, China; ^4^Shenzhen Academy of Inspection and Quarantine, Shenzhen 518010, China; ^5^School of Mechanical Engineering, Hebei University of Technology, Tianjin 300130, China; ^6^Department of Laboratory Medicine, Shenzhen Second People's Hospital, The First Affiliated Hospital of Shenzhen University, Health Science Center, Shenzhen 518035, China

## Abstract

**Objectives:**

We have developed a pulsed xenon ultraviolet light-based real-time air disinfection system with rapid and effective disinfection by using high-intensity pulse germicidal UV. Disinfection of the ambulance's environment is critical in the prevention of infectious cross contamination.

**Methods:**

In this study, a pulsed xenon ultraviolet light-based air disinfection system was established for real-time air disinfection in ambulances. In this system, a pulsed xenon ultraviolet (PX-UV) was used to generate broad-spectrum (200–320 nm), high-intensity ultraviolet light to deactivate and kill bacteria and viruses. The results showed that the use of PX-UV could be effective in reducing *E. coli*, *Staphylococcus albus*, and environmental pathogens level in ambulances (≥90% reduction in 30 mins).

**Results:**

This device was relatively simple and easy to use and does not leave chemical residues or risk exposing patients and workers to toxic chemicals.

**Conclusions:**

This appears to be a practical alternative technology to achieve automated air disinfection in ambulances.

## 1. Introduction

Hundreds of millions of patients around the world are affected by health care-associated infections (HCAI) each year, and despite the presence of many disinfection methods, microbial contamination remains a significant health concern throughout the world [1, 2]. The ambulance is one of the most common and important types of medical transport in the hospital emergency system. It is responsible for transferring individuals that are severely injured or ill. Because of the special construction and the narrow space inside, ambulances are frequently contaminated with pathogenic microorganisms shed by patients during prehospital transport, which would be transferred to subsequent patients and emergency medical service workers. Previous studies have demonstrated that ambulances operating in the emergency medical services (EMS) system may have a significant degree of MRSA contamination [3, 4]. These results demonstrated that ambulances represent an important reservoir for infectious microorganisms during an infectious disease pandemic, when large numbers of highly contagious patients would be transported. Disinfection of the ambulance's environment is critical in the prevention of infectious cross contamination. Chemicals such as chlorine dioxide and hydrogen peroxide disinfectant have traditionally been used for ambulance disinfection after used by infectious patients. However, manual chemical disinfection is tedious, time-consuming, and hazardous to the workers and the environment. Several studies have shown that persistent contamination is commonly found in patient compartment even after cleaning [5, 6]. During an infectious disease pandemic, a large number of highly contagious patients need to be transported and it requires ambulances return to service as soon as possible. In that case, separate routine cleaning is not sufficient to eliminate these pathogens. To control the cross infection in ambulances, good disinfection routines based on cleaning and disinfection of ambulances contaminated with highly contagious microorganisms are obligatory requirements.

Ultraviolet (UV) irradiation has been proposed as a terminal disinfection method in a variety of applications. The UV radiation covers the wavelength range from 100 to 380 nanometers. At certain wavelengths, the mechanism of killing of microorganisms by UV is primarily due to the breakage of the molecular bonds in DNA and RNA through absorption of photons resulting in formation of pyrimidine dimers from thymine and cytosine [7]. Specifically, previous studies discovered that UV radiation emitted at 254 nanometer (nm) was the most effective [8, 9]. Most UV room disinfection devices use mercury gas bulbs as a light source which has a characteristic wavelength of 254 nm [10]. The ultraviolet radiation emitted by low-pressure mercury bulbs is delivered in a continuous stream that gradually accumulates to lethal doses depending on the duration of exposure and distance from the primary field of radiation [11]. Pulsed xenon ultraviolet (PX-UV) is an attractive alternative to traditional UV methods offering high-intensity pulse germicidal UV. It is emitted in short, high-intensity pulses, possibly requiring a shorter duration of exposure to achieve lethal doses. PX-UV light may have greater efficacy than other forms of UV, such as mercury UV, because of the broad spectrum and a greater intensity [11]. Haddad et al. have shown that the use of PX-UV as a supplementing standard cleaning procedure helped reduce bacterial contamination levels [12]. Jinadatha et al. have shown that the use of PX-UV was more effective than standard manual room terminal cleaning in reducing levels of known pathogens [13–15]. To the best of our knowledge, a PV-UV-based disinfection device for real-time air disinfection in ambulances has not been previously reported.

The purpose of the current study was to validate a pulsed xenon ultraviolet disinfection device for real-time air disinfection in ambulances and to assess whether this device was effective in terms of reducing environmental pathogens contamination in ambulances.

## 2. Experimental Section

### 2.1. Device Setup

**Figure 1:**
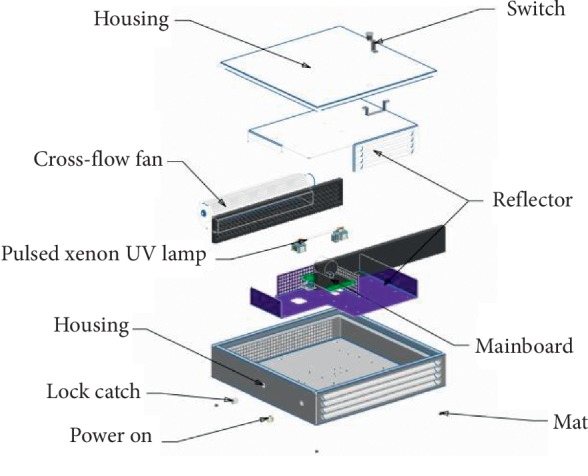
Experimental configuration of the pulsed xenon ultraviolet disinfection device (the size of the device is 505 × 500 × 120 mm).

The configuration of the real-time air disinfection device is shown in Figure 1. The unit is an enclosed air disinfection device incorporated into an ambulance fixture, where a pulsed xenon UV lamp is used as the light source, which can emit a broad spectrum of 200 nm–320 nm. The lamp was powered by a pulsed power source. The PX-UV system produces a pulsed flash at a frequency of 30 Hz with an approximate output of 270 J per pulse and a duration of less than 360 ms. The pulsed xenon UV lamp was placed in the center of a reflective, aluminum-covered chamber to continuously purify the air. The air flows through the processing chamber with an internal cross-flow fan with a flow rate of 5.4 m^3^/min. In this case, the cross-flow fan has two functions: (1) driving the air into the device and (2) cooling the pulsed xenon UV lamp. The reflectivity of the aluminum is proposed to enhance the light reflection efficiency and increase the time that the pulsed light is in contact with the air, thereby improving the germicidal activity of the apparatus. The air outlet was made in the form of shutters to block UV radiation.

### 2.2. Preparation of Bacterial Suspension

*E. coli* (ATCC 8099) and *Staphylococcus albus* (ATCC 8799) were used as the model bacteria to evaluate the sterilization effect. *E. coli* (ATCC 8099) and *Staphylococcus albus* (ATCC 8799) were obtained from Beijing Beina Chuanglian Biotechnology Institute and grown in the nutrient broth and nutrient agar at 36°C ± 1°C for 24 hours, followed by centrifugation at 3,300 ×g for 30 min. The bacteria were resuspended in 0.1 M phosphate buffer. A turbidimeter was used to prepare a bacterial suspension with a concentration of 1.5 × 10^8^ CFU/mL to 3.0 × 10^8^ CFU/mL. The prepared bacterial suspension will be ready for use.

### 2.3. Bacterial Suspension

The bacterial suspension was diluted with PBS buffer solution (the concentration of *E. coli* and *Staphylococcus* suspension was 1.20 × 10^6^ CFU/mL and 1.40 × 10^6^ CFU/mL, respectively). The diluted bacterial suspension was loaded in an aerosol generator (atomization effect >90%, particle size <5 *μ*m). This quasiexperimental study was conducted in two biosafety cabinets. The microbial aerosol generator was placed in biosafety cabinets (NUAIRE NU 437 600S). Air spray was carried out under the following conditions: the room temperature is 20°C to 25°C, and the relative humidity is 50% to 70%. The spraying time was 5 min and stationary for 1 min. The airborne bacterial populations were sampled by impaction directly onto nutrient agar plates, using a Merck MAS-100 air sampler. This was followed by the use of the PX-UV system, for 30-minute exposure. Replace the agar plate in the sampler and take the second sampling after 30 minutes. A control experiment was conducted as described above but without having been exposed to the PX-UV system. All plates were incubated at 36°C ± 1°C for 24 h.

(1) $$\begin{array}{c} N_{t}=\frac{V_{0}-V_{t}}{V_{0}}\times 100\%,\\ K_{t}=\frac{V_{0}^{\prime }\left(1-N_{t}\right)-V_{t}^{\prime }}{V_{0}^{\prime }\left(1-N_{t}\right)}\times 100\%, \end{array}$$

where *N*_*t*_ is the natural extinction rate of bacteria in the air, *V*_0_ and *V*_*t*_ are the amount of bacteria in the air at different times before and during the experiment was conducted, *V*_0_′ and *V*_*t*_′ are the amount of bacteria in the air at different times before and during the disinfection process of the experimental group, and *K*_*t*_ is the disinfection rate of bacteria in air.

### 2.4. Field Air Disinfection Test

To test the ability of the PX-UV system to disinfect pathogens in ambulances, we choose the ambulances that just returned to the hospital for their reasoned propensity to yield a large spectrum of bacteria. According to the ambulance instruction manual, the volume of the ambulance therapeutic cabin is approximately 10 m^3^. Before starting the disinfection device, a Merck MAS-100 air sampler was used to influence 1 L air onto blood agar plates before and after the pulsed xenon ultraviolet (PX-UV) disinfection for 60 minutes. All the plates incubated at 36°C ± 1°C for 24 h. All bacteria and fungi colony forming units were counted, and the airborne bacterial count and killing rate were calculated:

(2) $$\begin{array}{c} \text{Killing rate}\left(\%\right)=\frac{\text{Bacterial count before disinfection}-\text{bacterial count after disinfection}}{\text{Bacterial count before disinfection}}. \end{array}$$

## 3. Result and Discussion

In this study, we used *E. coli* and *Staphylococcus albus* as models to test the disinfection effect of PX-UV. Tables 1 and 2 show the *E. coli* and *Staphylococcus albus* concentration levels before and after the PX-UV treatment, respectively. It can be seen that the 30-min PX-UV treatment reduces the *E. coli* concentration which is lower than the detection level, while the PX-UV treatment results in 99.91% *Staphylococcus albus* disinfection. UV can kill bacteria, viruses, fungi, and spores, but different types of microorganisms have different sensitivity to UV, Gram-negative bacteria are the most sensitive, followed by *Staphylococcus* [16]. The available reason for this case is that *E. coli* is more sensitive to UV light than *Staphylococcus albus*. Therefore, it is seen that PX-UV treatment for more than 30 mins has an apparent effect in reducing the bacteria concentration levels to a value compatible with the guidelines.

The disinfection efficiency of the real-time PX-UV disinfection device was evaluated by measuring the bioaerosol levels of natural bacteria before and after disinfection. The experimental results found that the average disinfection rate of natural bacterial aerosols was found to be more than 90% after 60 mins of disinfection, which was lower than that of the laboratory simulation test (see Table 3). Because of the harsh living conditions in the natural environment, the survival ability of the living microorganisms and the ability to resist external interference are stronger than those used in the laboratory. Thus, using a real-time PX-UV disinfectant to maintain the air quality is of great importance to reduce cross infection in ambulances.

## 4. Conclusions

In conclusion, we have developed a pulsed xenon ultraviolet light-based real-time air disinfection system with rapid and effective disinfection by using high-intensity pulse germicidal UV. Our design is an enclosed air disinfection device, which can be operated in the presence of people. The device is powered by an ambulance and can be operated automatically as the ambulance starts.

In our study, we found that the real-time air disinfection device reduced the number of *E. coli* and *Staphylococcus albus* on the biological safety cabinet with a 30-minute exposed time and foresaw a positive effect. Similarly, because of the complex environment of the actual site, only 90% of the bactericidal results have been achieved. Although the disinfection effect has not reached more than 99%, the efficacy of the real-time air disinfection device could get the desired results in real-world settings. The results of this study suggest that the home-made PX-UV disinfection device can provide real-time and effective disinfection for ambulance application.

In actual use, some problems with the real-time air disinfection device have been discovered. The first is the ventilation problem. The space for an ambulance is approximately 10 m^3^. Does the air in the ten cubic meters of space is completely sterilized by the device instead of being sterilized clean air all the time? Secondly, the contact surface of the equipment considering the disinfection air needs to be large enough and the shape of the designed instrument needs further improvement. The space in the cabin is limited, with excellent disinfection effects while saving space as much as possible, so that the medical staff can have enough space to treat patients. For these problems, we also need to further design and improve the instrument to produce a better device to achieve higher and more effective disinfection.

## Figures and Tables

**Table 1:** Effectiveness of the real-time air disinfection device against *E. coli* in the air.

Time (min)	No.	*V*_0_ (Cfu/m^3^)	*V*_*t*_ (Cfu/m^3^)	*N*_*t*_ (%)	*V*_*o*_′ (Cfu/m^3^)	*V*_*t*_′ (Cfu/m^3^)	*K*_*t*_ (%)
30	1	2.70 × 10^5^	1.76 × 10^5^	34.81	2.64 × 10^5^	0	100
	2	2.54 × 10^5^	1.66 × 10^5^	34.65	2.40 × 10^5^	0	100
	3	2.10 × 10^5^	1.40 × 10^5^	33.33	2.10 × 10^4^	0	100

*N*_*t*_: the natural extinction rate of bacteria in the air; *V*_0_ and *V*_*t*_: the amount of bacteria in the air at different times before and during the experiment was conducted; *V*_*o*_′ and *V*_*t*_′: the amount of bacteria in the air at different times before and during the disinfection process of the experimental group; *K*_*t*_: the disinfection rate of bacteria in air.

**Table 2:** Effectiveness of the real-time air disinfection device against *Staphylococcus albus* in the air.

Time (min)	No.	*V*_0_ (Cfu/m^3^)	*V*_*t*_ (Cfu/m^3^)	*N*_*t*_ (%)	*V*_*o*_′ (Cfu/m^3^)	*V*_*t*_′ (Cfu/m^3^)	*K*_*t*_ (%)
30	1	2.64 × 10^5^	1.76 × 10^5^	33.33	2.64 × 10^5^	160	99.91
	2	2.44 × 10^5^	1.51 × 10^5^	38.11	2.52 × 10^5^	150	99.90
	3	2.31 × 10^5^	1.40 × 10^5^	39.39	2.40 × 10^5^	130	99.91

*N*_*t*_: the natural extinction rate of bacteria in the air; *V*_0_ and *V*_*t*_: the amount of bacteria in the air at different times before and during the experiment was conducted; *V*_*o*_′ and *V*_*t*_′: the amount of bacteria in the air at different times before and during the disinfection process of the experimental group; *K*_*t*_: the disinfection rate of bacteria in air.

**Table 3:** Disinfection efficiency of the real-time PX-UV for natural bacteria in ambulances.

Time (min)	No.	Before (Cfu/m^3^)	After (Cfu/m^3^)	Killing rate (%)
60	1	480	30	93.75
	2	490	40	91.84
	3	400	40	90.00

## Data Availability

No data were used to support this study.
